# Supplementary material for: Technology-driven reduction of fish post-harvest loss could enhance food security and economic resilience
Source: Commun Sustain. 2026 Mar 11;1(1):45. doi: 10.1038/s44458-026-00048-4 (PMC12979193; doi:10.1038/s44458-026-00048-4)
Supplement: Supplementary file 2 — Supplementary Table 1 [file 44458_2026_48_MOESM2_ESM.pdf]

Supplementary Table 1. Continental parameters used in the post-harvest utilisation model.

	a_0	lim_a	b_0	lim_b	c	d_0	d_{max}
Africa	81%	55%	72%	56%	65%	15%	70%
Asia	92%	70%	77%	56%	65%	35%	70%
Europe	87%	75%	85%	56%	65%	40%	70%
North America	86%	75%	81%	56%	65%	50%	70%
Oceania	74%	70%	75%	56%	65%	20%	70%
South America	73%	55%	82%	56%	65%	30%	70%
World	89%	68%	81%	56%	65%	30%	70%
